# Ridge Regression in Prediction Problems: Automatic Choice of the Ridge Parameter

**DOI:** 10.1002/gepi.21750

**Published:** 2013-07-26

**Authors:** Erika Cule, Maria De Iorio

**Affiliations:** 1 Department of Epidemiology and Biostatistics, Imperial College LondonLondon, United Kingdom; 2 Statistical Consulting Group, GlaxoSmithKlineStevenage, United Kingdom; 3 Department of Statistical Science, University College LondonLondon, United Kingdom

**Keywords:** GWAS, penalised regression, prediction, shrinkage methods, software

## Abstract

To date, numerous genetic variants have been identified as associated with diverse phenotypic traits. However, identified associations generally explain only a small proportion of trait heritability and the predictive power of models incorporating only known-associated variants has been small. Multiple regression is a popular framework in which to consider the joint effect of many genetic variants simultaneously. Ordinary multiple regression is seldom appropriate in the context of genetic data, due to the high dimensionality of the data and the correlation structure among the predictors. There has been a resurgence of interest in the use of penalised regression techniques to circumvent these difficulties. In this paper, we focus on ridge regression, a penalised regression approach that has been shown to offer good performance in multivariate prediction problems. One challenge in the application of ridge regression is the choice of the ridge parameter that controls the amount of shrinkage of the regression coefficients. We present a method to determine the ridge parameter based on the data, with the aim of good performance in high-dimensional prediction problems. We establish a theoretical justification for our approach, and demonstrate its performance on simulated genetic data and on a real data example. Fitting a ridge regression model to hundreds of thousands to millions of genetic variants simultaneously presents computational challenges. We have developed an R package, ridge, which addresses these issues. Ridge implements the automatic choice of ridge parameter presented in this paper, and is freely available from CRAN.

## Introduction

In modern biomedical studies, technological developments mean that data sets are now much larger than those considered in the past. The analysis of these high-dimensional data sets presents computational and statistical challenges. Specifically in genetics, genome-wide single nucleotide polymorphism (SNP) genotyping simultaneously types up to one million SNPs, and imputation can increase SNP density further. In the related fields of metabolomics and proteomics, information is captured about hundreds of thousands or millions of variables.

In this paper, we are interested in the use of genetic information to predict either a continuous or a binary phenotype. Both of these outcome types arise in clinical settings. In the latter case, we consider both the problem of classification and the estimation of the probability of each of the two possible outcomes. We evaluate our method using SNP genotype data; however, the methods discussed here could usefully be applied to any high-dimensional regression setting.

### Genetic Risk Prediction

Recent developments and improvements in genotyping technology have led to an increase in the availability of genetic information. As genotyping becomes easier and cheaper, there is a growing interest in the use of genetic information to predict future phenotypes, and in particular in the prediction of disease phenotypes. The aim of genetic prediction is to construct a model to predict the phenotype of interest using the genotype data from individuals for whom the (potentially future) phenotypic state is unknown. In a clinical setting, the value of this approach arises because genotypes are fixed at birth, but disease symptoms may not become apparent until later in life. If individuals who are at high genetic risk could be identified before symptoms become apparent, targeted interventions could be used with the aim of delaying or preventing the onset of disease. Drug response is known to be genetically heterogeneous [Goldstein, 2005], and the ability to predict drug response based on genetic information would also be valuable as it would enable the tailoring of optimally effective treatments.

Genome-wide association studies in particular have led to the identification of numerous genetic variants as associated with diverse phenotypic presentations [Hindorff et al., 2009]. Existing approaches that use genetic information to predict disease risk have focussed on the exploitation of these established genetic associations. In these risk prediction models, genetic variants that have been identified as associated with the phenotype of interest in previous association studies are incorporated into a risk prediction model. A simple count of risk variants may be used, or each variant may be weighted by the strength of the association, measured for example using the log-odds ratio. Associated risk variants may be incorporated into the risk prediction model instead of or in addition to clinical risk factors such as sex, age or family history.

Complex traits often exhibit moderate to high heritability. However, genetic variants identified in association studies as associated with complex traits often combine to explain only a small proportion of the overall heritability of that trait [Eichler et al., 2010]. In prediction problems, when identified associated genetic variants are incorporated in risk prediction models, either instead of or in addition to established clinical risk factors, overall predictive performance has been disappointing. The inclusion of genetic risk factors offers little or no improvement in disease risk prediction compared to clinical risk factors alone. A plausible hypothesis for the genetic architecture of complex diseases is that the genetic contribution results from many risk variants each with small effect size. Therefore, interest has turned to the development of prediction models that incorporate many more genetic variants, up to and including all typed variants in a whole-genome regression. Genotype data are high-dimensional, and usually consist of many more predictors than observations. Further, genetic variants can be highly correlated due to linkage disequilibrium. These properties of genetic data mean that traditional multiple regression approaches cannot be applied. There has been a growing interest in the use of penalised regression approaches to the analysis of genetic data [Abraham et al., 2013; Ayers and Cordell, 2010]. Ridge regression (RR) is one such penalised regression approach.

RR was originally proposed as a means of estimating regression coefficients with smaller mean-square error than their least squares counterparts when predictors are correlated [Hoerl and Kennard, 1970]. RR is one of a family of penalised regression methods, other popular methods include Lasso regression [Tibshirani, 1996] and the Elastic Net [Zou and Hastie, 2005b]. The latter is a weighted combination of the Lasso and ridge penalties. Among penalised regression approaches, RR has been shown to offer good predictive performance [Frank and Friedman, 1993]. One challenge in applying penalised regression methods is the choice of the shrinkage parameter, or parameters, which control the amount of shrinkage of the regression coefficients. In RR, a number of numerical and data-driven methods have been proposed, but no consensus method provides a universally optimum choice. It is the problem of the choice of the shrinkage parameter in RR for high-dimensional data that we address in this paper.

In a comparative study, Abraham et al. [2013] compared penalised and unpenalised regression methods for prediction in complex disease. There, the authors found that the sparse penalised methods that they considered, Lasso and Elastic Net regression, outperformed other methods for prediction in diverse disease phenotypes. However, Abraham et al. did not address the problem of the choice of shrinkage parameter or parameters in the penalised regression methods that they used, nor did was RR among the methods that they compared.

The improvement in heritability estimates that can be achieved when all genetic variants, whether associated with the phenotype of interest or not, are included in the study, was demonstrated by Yang et al. [2010]. In an investigation into the heritability of complex traits, Yang et al. demonstrated that in a population of unrelated individuals, 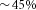 of the variance in human height could be explained by considering a genome-wide panel of nearly 300,000 common SNPs. This represents a substantial increase over the 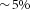 of phenotypic variance explained by the ∼50 variants known to be associated with human height. This provides support for our notion that the inclusion of many more SNPs in a prediction model will improve predictive performance. However, Makowsky et al. [2011] cautioned that the increased proportion of variance explained using genome-wide SNP data did not translate into validation samples.

Accounting for linkage disequilibrium among SNPs in genome-wide association studies using penalised regression has been investigated both in variable selection [Malo et al., 2008] and in classification problems [Malovini et al., 2012]. Malo et al. [2008] compared RR with SNP-by-SNP variable selection and standard multiple regression and found that RR outperformed the unpenalised methods in detecting causally related variables. Malovini et al. [2012] compared a Hierarchical Naive Bayes classifier (HNB), in which SNPs in LD are considered to be a single latent variable, to a Naive Bayes classifier (NB) that assumes independence among the predictors. They found that the HNB performed equally well or better than NB, and that HNB was particularly advantageous when analysing genomic regions in strong LD.

RR and other penalised regression methods have been studied in the animal and plant breeding literature, where they are used to predict breeding values based on genetic markers [Meuwissen et al., 2001]. There, marker panels are typically smaller than the number of SNPs on a human GWAS SNP chip, and tuning parameters are often chosen by cross-validation [Moser et al., 2009]. A review of penalised regression methods used in genomic prediction, from both frequentist and Bayesian perspectives, can be found in de los Campos et al. [2013]. In this paper, our aim is to make RR feasible and useful for risk prediction using genome-wide SNP data.

We propose a semi-automatic method to guide the choice of ridge parameter in RR. Our proposed method is valid when the regression problem comprises more predictors than observations, and is based on an established technique, that of Hoerl et al. [1975].

## Methods

### Summary of the Proposed Method

We consider data on *n* individuals, 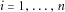, where each individual has been genotyped at *p* loci, 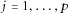. Then, **X** is the 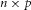 matrix of genotyped variants, standardised such that 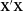 is in correlation form. Typically, as in this study, the genetic variants are SNPs, but other types of variation could be used. 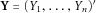 is a *n*-dimensional vector of phenotype measurements that have been centred to have mean 0. When phenotypes are continuous, the linear regression model is commonly used to model the relationship between genotypes and a phenotype of interest:

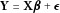
1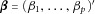 is a column vector of *p* regression coefficients, one for each genotyped variant, and 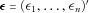 is a vector of independent and identically distributed normally distributed random errors, 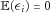 and 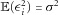. Ridge estimates of the regression coefficients are given by

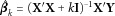
2*k* is the shrinkage parameter, which controls the amount of shrinkage of the regression coefficients, and **I** is the identity matrix. Below, we summarise the method we propose for determining the shrinkage parameter based on the data. We denote this shrinkage parameter 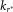. A theoretical justification for our proposed approach is presented at the end of this Methods section. Readers who are not interested in the technical details of our method can skip to the simulation study that follows.

### Algorithm to Determine 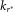

1. Calculate the eigenvectors and eigenvalues of 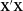:

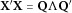
Here, columns of **Q** are the eigenvectors of 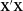 and 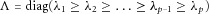 is a diagonal matrix with diagonal elements the eigenvalues of 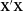 in descending order. Of the *p* diagonal elements of Λ, at most 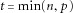 are non-zero.
2. Compute the principal components of **X** as 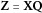, and the principal components regression (PCR) coefficients as

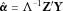
3
3. For 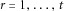, compute 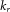 as

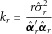
where 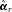 is the *r*-length vector of the first *r* principal components regression coefficients, 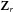 are the first *r* columns of **Z**, and

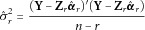
Among possible *r*, choose 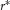 as the value of *r* that minimises

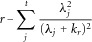

4. Denote 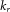 using the chosen value of *r* as 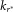 and use this in fitting the ridge estimates:

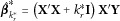

The motivation behind the method we propose is to choose a shrinkage parameter that performs at least as well as, and often better than, a principal components regression model with the same degrees of freedom in prediction problems. Our method has the advantage of improve interpretability of the fitted coefficients relating genetic variants to phenotype.

### Adapted algorithm for binary traits

Binary outcomes often arise in biomedical studies, where they represent, for example, disease cases and healthy controls. For binary traits we use an adapted version of the previous algorithm, based on the logistic regression model, as follows:

1. Calculate the principal components of **X** as in steps 1–2 of the algorithm for continuous traits.
2. For 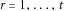, compute 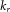 as

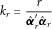
Here, 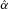 is the *r*-length vector of the first *r* principal components logistic regression (PCLR) coefficients, as in Aguilera et al. [2006]. Briefly, PCLR coefficients are computed using a subset of the principal components of the predictors as covariates in a logistic regression model.
3. Calculate the degrees of freedom of the ridge logistic regression model fitted using 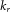 as the shrinkage parameter, and choose the number of PCs, 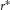, to minimise the difference between *r* and the degrees of freedom of the corresponding fitted ridge logistic regression model.
4. Use the corresponding shrinkage parameter 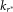 in a ridge logistic regression model fitted on the full data set in its original orientation.

Details of ridge logistic regression are given in the next section and in Supplementary Appendix C.

### Theoretical Justification

The method we propose is developed based on a popular choice of the ridge parameter that was proposed by [Hoerl et al., 1975], which we denote *k*_HKB_ after the authors who proposed it. Here, 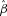 are the ordinary least squares (OLS) regression coefficients, equivalent to equation 2 with 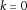. Hoerl et al. proposed the following choice of ridge parameter:

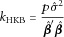
4where

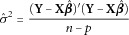
When 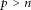, *k*_HKB_ is not defined, because it is dependent on the ordinary least squares regression (OLSR) coefficients 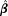 that are themselves not defined when the data have more predictors than observations. When 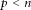, *k*_HKB_ can equivalently be written

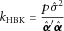
In a generalised RR, individual shrinkage parameters are assigned to each of the principal components in the linear model. Then, *k*_HKB_ is motivated as the harmonic mean of the ‘ideal’ generalised ridge estimator in terms of minimising mean square error of the coefficient estimates. Hoerl et al. explain their choice of the harmonic mean as a way to preventing the small 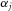, which have little predicting power, leading to too large a a shrinkage parameter as would happen if the arithmetic mean were to be used. We observed that 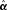 in this estimator are the principal components regression coefficients defined in equation 3. In a simulation study with 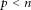, we investigated the use of 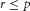 principal components in computing 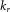. In Supplementary Appendix A and Supplementary Fig. S1 we demonstrate that when the signal-to-noise ratio is not too low, estimates of 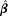 with smaller mean squared error are obtained using 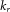 with 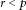 than when using *k*_HKB_.

With evidence that using 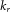 with 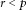 as a shrinkage estimator can result in improved estimates of 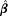 compared to when *k*_HKB_ is used, our method is naturally extensible to data with more predictors than observations (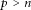). We need to determine whether inclusion of all nonzero PCs results in estimates with smallest prediction error or whether smaller prediction error is obtained when using the first *r* PCs, 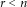. We evaluated this using simulation studies. The details of the simulations are given in the Simulation Study section, where we evaluate the performance of RR using 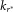 in comparison to other penalised regression methods. 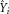 is the predicted phenotype of the *i*^th^ individual using the fitted coefficients. Where data have continuous outcomes, we evaluated performance in terms of mean prediction squared error (PSE).

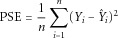
5

Where data have binary outcomes, we used mean classification error (CE), as in Le Cessie and Houwelingen [1992]:

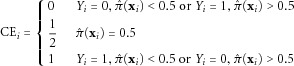
6Here, 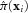 is the estimated probability that the *i*^th^ individual is a case based on his genotypes, that is 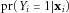. We take the average CE:

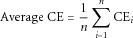
7

In Table1, we report average PSE when 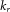 is calculated using different numbers of PCs, in simulated genetic data with more predictors than observations. In a principal component decomposition, PCs are arranged by the decreasing amount of overall variability explained by each component. However, in each simulation replicate, the amount of overall variability in the predictors explained by each PC will vary due to the different correlation structure among the predictors. To be able to summarise results across simulation replicates, instead of comparing results at different values of *r*, we calculate the proportion of variance explained by PCs included in the calculation of 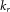 and use this when summarising predictive performance across simulation replicates. We see that minimum PSE is obtained when fewer than the maximum number of PCs are used to compute 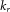. Table1 shows prediction squared error or classification error at different proportions of variance explained. MAX: *r* is the maximum number of PCs where the corresponding eigenvalues are nonzero. CV: 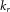 with *r* chosen using the cross-validated PRESS statistic [De Iorio et al., 2008]; 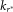 is as described previously. We see from columns 1–5 of Table1 that the best predictive performance is obtained when somewhat fewer than the maximum number of PCs is used to compute 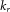. Of the rules we investigated, 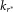 offers marginally best predictive performance.

**Table 1:** Prediction squared error (PSE) or classification squared error (CSE) in out-of-sample prediction using *r* with different proportions of variance explained

	Proportion of variance explained by PCs used to compute 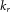 (%)					RR parameter	
	10	50	70	90	MAX	CV	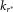
Continuous outcomes (mean PSE)	1.24	1.23	1.23	1.27	3.20	1.24	1.23
(sd)	(0.06)	(0.05)	(0.05)	(0.06)	(0.87)	(0.05)	(0.05)
Binary outcomes (mean CE)	0.46	0.47	0.47	0.47	0.47	0.47	0.46
(sd)	(0.03)	(0.03)	(0.03)	(0.04)	(0.03)	(0.03)	(0.03)

MAX, *r* is the maximum number of PCs where the corresponding eigenvalues are non-zero; CV, *r* chosen using cross-validated PRESS statistic; 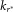: *r* chosen based on degrees of freedom (see main text); SD, standard deviation.

With evidence from Table1 that computing 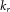 using 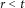 gives rise to coefficient estimates with smaller prediction error than when the maximum number of PCs is used, we use the following arguments to justify our rule (see step 3 of the Algorithm presented earlier) to determine the number of components, 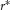, to use in computing 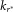. Regression models including PCR, RR and OLSR all result in models in which the fitted outcomes can be related to the observed ones via a projection matrix or ‘hat’ matrix:

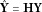
8In OLSR, RR and PCR, **H** is of the form

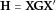
9The definition of **G**, and thus **H**, depends on the model being fitted. In OLSR, 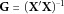 and 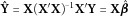. Both RR and PCR use **G** that approximates 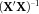 in a different way [Brown, 1993]. In RR, 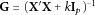 where *k* is the ridge parameter and 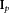 is the *p*-dimensional identity matrix.

In PCR, **G** is given by

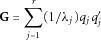
where 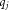 is the *j*^th^ column of **Q**. For models that can be written in this form, PSE can be decomposed as:

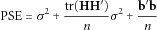
10where 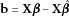 is the bias, the distance between the fitted estimates and the true ones. The first term measures the (unavoidable) noise in **Y**, the second measures variance in the prediction estimates. In Supplementary Appendix B, we discuss tr(**H**) and 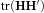 as definitions of effective degrees of freedom for the model and for variance, respectively. There, we show that among different definitions of degrees of freedom, using 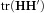 in computing 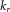 results in coefficient estimates closest to the OLS estimates.

In OLSR, 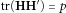, the number of parameters in the regression fit. The aim in penalised regression is to reduce the variance by allowing the introduction of a little bias, keeping the overall PSE lower than that of OLSR. In PCR, 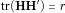, the number of components used in the penalised regression fit.

In RR, it is straightforward to find a shrinkage parameter *k* such that 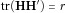 where *r* is any specified value, by noting that 
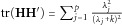
 and using numerical methods to find *k*. Thus, we can compare PCR and RR in terms of prediction squared error, equation 10, when the variance in the prediction estimates is forced to be equal to *r* in both models. With common error variance σ^2^ across models, we are only interested in the bias term and we can find an expression for this also, by noting that:

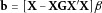
11

In OLSR, the estimates are unbiased (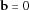). In a simulation study with 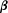 known, we can compare the bias in RR and PCR when the variance of the fitted 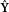 in each model is fixed such that 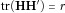.

In PCR, the coefficients of the first *r* PCs are their least squares counterparts; the coefficients of the remaining components are set to zero. Thus the bias is the difference between zero and the least squares estimate of the coefficient of the 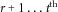 components, where 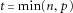 is the maximum number of PCs. In RR the bias is more ‘spread out’ among the *t* components as the least squares estimate of each coefficient is ‘shrunk’ by 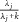.

We illustrate this using a simulation study. The patterns of predictors and coefficients used by Zou and Hastie [2005a] are used here. Although these are not genetic data, they do cover a range of parameter values and correlation structures, enabling us to illustrate the bias-variance decomposition of the PSE. The four regression scenarios are detailed in Supplementary Table S1.

**Figure 1:**
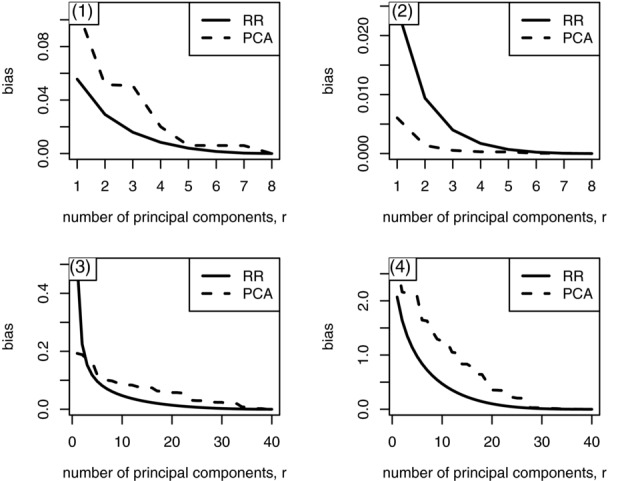
Bias 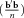 in PCR and RR in regression scenarios (1), (2), (3), and (4) (Supplementary Table S1), at different values of *r*.

In Figure 1, we plot 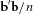 using **b** defined as in equation 11 for 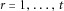. We see that for regression scenarios (1), (3) and (4) in Supplementary Table S1, the bias is typically lower for RR than for PCA. The only scenario in which PCA has lower bias than RR is scenario (2), where there is moderate correlation among the predictors but all the coefficients have the same effect size, a situation that is unlikely to arise in genetic data. We can see the smooth decrease in the bias with RR whereas in PCR the bias decreases in a stepwise manner, with each step corresponding to the inclusion of one more component in the model. As *r* approaches *t*, the fitted coefficients approach their least squares counterparts and the bias approaches 0, its value in OLSR.

**Figure 2:**
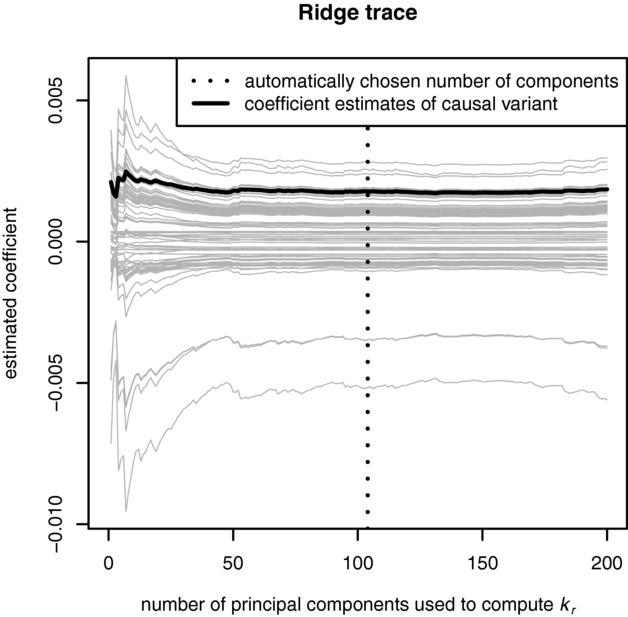
Ridge trace showing estimated regression coefficients estimated using 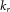 computed using different numbers of PCs. The *x*-axis shows the number of PCs used to compute 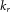. The vertical dotted line indicates that our proposed method of choosing the number of components chooses a ridge parameter in the region where ridge estimates stabilise. The black line indicates a causal variant. Plotted are the first 100 SNPs of the 20,000 in one simulation replicate, with continuous outcomes.

In Figure 2 we plot the fitted RR coefficients with different values of *r* used to compute 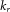. These plots are taken from one simulation replicate with continuous outcomes. 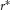, the number of components that minimises 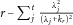 is indicated and a causal variant is highlighted. We see that choosing *r* to minimise 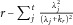 results in a shrinkage parameter in a region of the ridge trace where the RR coefficients are stable and do not change much with further increases in the number of principal components. We evaluate this rule in comparison to competing methods using simulation studies in the next section.

The theoretical justification for our method in the case of binary traits derives from the use of ridge logistic regression (as opposed to linear RR). The logistic regression model is commonly used to model the effect of one or more predictors on a binary response. When predictors are highly correlated or high-dimensional, maximum likelihood estimation of logistic regression coefficients may have large variance. When predictors are exactly correlated, or there are more predictors than observations, maximum likelihood estimates are not defined.

Ridge logistic regression has been proposed as a means of addressing these difficulties. Schaefer et al. [1984] introduced a ‘Ridge type’ estimator and demonstrated that, when predictor variables are collinear, this will result in coefficient estimates with smaller mean squared error than the maximum likelihood estimates. The ‘Ridge type’ estimator proposed by Schaefer et al. [1984] is

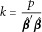
12Here, 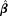 are maximum likelihood estimates of the logistic regression coefficients. We extended the approach that we used to compute 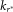 in linear RR to logistic RR. We use increasing numbers of PCs in a PCLR [Aguilera et al., 2006] to compute the shrinkage parameter. For a PCLR using *r* components, the corresponding penalty is calculated as

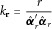
where 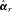 is the vector of *r* regression coefficients computed using PCLR. In both linear and logistic RR, the number of components used to compute the shrinkage parameter is based on the degrees of freedom of the regression fit. To estimate the effective degrees of freedom of the logistic regression model, we used the trace of the square of the hat matrix, which in the logistic RR model is calculated as:

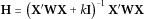
where 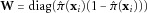. 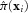 are the fitted probabilities. These are the probabilities under the logistic model, computed using the fitted coefficients obtained at the last iteration of the optimisation algorithm. Details of logistic regression are discussed in Supplementary Appendix C. As in linear RR, we compute the effective degrees of freedom for variance as 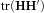.

## Simulation Study

We used simulated data to compare the predictive performance of RR models fitted using 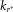 to that of competing regression approaches that can be applied to high-dimensional data.

### Simulated Data

Simulated SNP data were generated using the software FREGENE [Chadeau-Hyam et al., 2008; Hoggart et al., 2007] as a panmictic population of 21,000 haplotypes. We used a region of approximately 7 Mb, containing 20,000 SNPs with minor allele frequency (MAF) 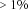. FREGENE simulates the evolution of haplotypes forward-in-time, with mutation and selection parameters that can be specified when the program is run. The simulated data we used are available for download (http://www.ebi.ac.uk/projects/BARGEN/) and details of the simulation are described in Chadeau-Hyam et al. [2008]. Genotypes are coded as 0, 1, 2 for minor allele count.

Genotype and corresponding phenotype data were simulated with both continuous and binary outcomes, analysed using linear and logistic regression, respectively. Each replicate consisted of 1,000 training individuals and 500 test individuals, and results were averaged over 10 replicates. Causal SNPs were selected from those SNPs in the MAF range 10–15%, following the common-disease common-variant hypothesis for complex diseases. Data were generated and analysed as follows:

- Continuous outcomes analysed using linear regression. Two hundreds SNPs with MAF 10–15% were randomly selected to be causal SNPs; these causal SNPs were assigned an effect size drawn from a uniform distribution *U*[0.05, 0.1]. All other SNPs were given an effect size of 0. Thus the vector of effect sizes, 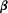, of length 20,000, contained 200 non-zero elements. Genotypes were generated by summing two randomly selected haplotypes. An additive genetic model was assumed. Following such a model, responses were generated as 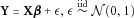. Model performance was evaluated using PSE, equation 5, over the test data.
- Binary outcomes analysed using the logistic model. Two hundreds SNPs with MAF 10–15% were randomly selected to be causal SNPs; these causal SNPs were assigned an effect size, or log odds ratio, drawn from a uniform distribution *U*[0.1, 0.5]. Case-control outcomes were generated by randomly selecting two haplotypes that were summed to generate a simulated genotype. An additive genetic model was assumed. Following such a model, the probability of an individual with that genotype being a case was generated as 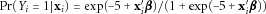, and that individual's case-control status was determined randomly according to this probability. This process was repeated until the required (equal) number of cases and controls was obtained. For the data with binary outcomes, predictive performance is reported in two ways. First, we use classification error, as in equation 6. Second, we report the Brier score [Brier, 1950], a measure of the accuracy of the predictions. The Brier score takes values between 0 and 1, with smaller values indicating more accurate predictions. The reported Brier score is the average over the 10 replicates.

In both the simulation studies and the evaluation of the method using real data that follows, training genotypes were standardised to correlation form, and in the continuous case the responses were centred, prior to model fitting, as described in methods section. Coefficients were returned to the scale of the original data when the model was evaluated on test data.

### Simulation Study Results

We compared the performance of RR using 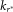 to four competing methods of fitting prediction models to high dimensional data:

1. We used traditional multiple linear or logistic regression, which requires that a subset of SNPs be selected for inclusion in the model. To select which SNPs to include, univariate linear or logistic regression was used to estimate the strength of association of each predictor variable with the outcome. A proportion of the predictor variables that were most strongly associated with the outcome in univariate tests were included in a multiple regression model. When predictors were exactly correlated and both predictors cannot be included in a multiple regression model simultaneously, only the first predictor by SNP position was included. Because in real applications the number of causal variables is not known *a priori*, we evaluated the predictive performance when a range of proportions of predictors were included in the multiple regression.
2. RR with the ridge parameter chosen using 10-fold cross-validation (RR-CV).
3. HyperLasso regression (HL, Hoggart et al. [2008]) is a penalised regression method that simultaneously considers all predictor variables in a high-dimensional regression problem. HyperLasso was originally applied to the problem of identifying causal variables when predictors are correlated, but it was shown that by using a less stringent threshold for inclusion of predictors in the model, HyperLasso could be used to address the problem of prediction. In order to obtain good predictive performance, it is necessary to relax the penalty so that sufficient coefficients are estimated as non-zero. The penalty in HyperLasso regression is such that, among a group of correlated predictors, only one coefficient will be estimated as non-zero. This is a disadvantage in prediction using genetic data, where several correlated predictors, for example in one LD block, may contain information that is useful for prediction even if if they are not all causal variables. HyperLasso requires the specification of two parameters to control the amount of shrinkage. Following Eleftherohorinou et al. [2009] the shape parameter in HyperLasso regression was fixed to 3.5 and the penalty parameter was chosen using 10-fold cross-validation. The penalised coefficients fitted using the parameters chosen using cross-validation were used to evaluate prediction performance.
4. Elastic net (EN, Zou and Hastie [2005b]) is a penalised regression method that combines the ridge and Lasso penalties. EN requires two parameters, one to control the relative weights of the ridge and Lasso penalties and one to control the amount of shrinkage. EN models were fitted using the R package glmnet [Friedman et al., 2010] and the parameters were chosen using 10-fold cross-validation.

Results are presented in Table2. In this regression problem, which is a realistic simulation of risk prediction in genetic data, RR outperforms the competing penalised regression methods, EN and HL. Using univariate variable selection followed by multiple regression, the best performance was obtained when the number of predictors included in the model was equal to the number of non-zero regression coefficients when the data were generated, a proportion that would not be known in practice. In HyperLasso regression, CV to choose the penalty parameter was computationally demanding. We found in order to obtain the best predictive performance a relaxed penalty was necessary. CV to choose the parameters for the elastic net showed that that more than half of the time the alpha parameter was set to zero, which makes EN equivalent to RR [Friedman et al., 2010]. The CV choice of the parameter that controls the amount of shrinkage of the regression coefficients resulted in a large parameter being chosen, implying strong shrinkage. Together, the CV choice of EN parameters results in the equivalent of an RR with the regression coefficients shrunk close to zero. This reflects the structure of the data with many predictors of small effect. RR-CV and RR-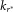 offer approximately equivalent predictive performance. Although 10-fold CV is feasible for a data set of this size, fitting an RR model on GW data (as in the real data example that follows) using the coordinate descent algorithm (see Supplementary Appendix C) took so long as to make cross-validation on GW data infeasible. (On an iMac with a 2.8 GHz Intel Core i7 processor and 16 GB of RAM, running R 2.15.1 (64-bit) and GSL-1.14 fitting an RR model to genome-wide SNP data consisting SNP data on 4,727 individuals at 336,044 SNPs took approximately 20 h.) Thus the method we propose is computationally more straightforward than competing penalised regression methods, is applicable when the number of predictors exceeds the number of observations, and offers predictive performance that matches that of 10-fold CV, whilst being computationally feasible for genome-wide SNP data. In simulated data with binary outcomes, we report the Brier score as a measure of prediction accuracy. Again we see that RR outperforms the competing methods, and we reiterate the reduced computational time taken to choose the ridge parameter using our automatic method compared to using cross-validation.

**Table 2:** Performance in out-of-sample prediction in simulated data using different methods to fit prediction models

	Univariate					RR-CV	HL	EN	RR- 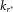
% of SNPs ranked by univariate *P*-value	0.1%	0.5%	1%	3%	4%				
Continuous outcomes (mean PSE)	1.51	1.55	1.54	2.21	3.93	1.22	2.41	3.26	1.23
(sd)	(0.10)	(0.11)	(0.11)	(0.54)	(1.34)	(0.05)	(0.31)	(0.21)	(0.05)
Binary outcomes (mean CE)	0.49	0.48	0.49	0.49	0.49	0.46	0.50	0.48	0.46
(sd)	(0.03)	(0.03)	(0.03)	(0.03)	(0.01)	(0.03)	(0.03)	(0.04)	(0.03)
Binary outcomes (Brier score)	0.26	0.28	0.31	0.37	0.41	0.25	0.30	0.27	0.25
(sd)	(0.01)	(0.02)	(0.06)	(0.06)	(0.05)	(0.005)	(0.06)	(0.04)	(0.003)

RR-CV, RR with the shrinkage parameter chosen using 10-fold cross-validation; HL, HyperLasso; EN, Elastic Net; RR-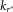, RR with the shrinkage parameter 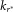.

## Implementation

We have developed an R package, ridge, which implements our method (available from CRAN: http://cran. r- project. org/web/packages/ridge). This package addresses the computational challenges that arise when fitting RR models to high-dimensional data such as genome-wide SNP data. For data sets that are too large to read into R, code written in C is provided and the corresponding R functions take file names as arguments. This circumvents the need to read large data sets into the R workspace whilst retaining a user-friendly interface to the code.

Logistic RR is performed using the CLG algorithm [Genkin et al., 2007]. CLG is a cyclic coordinate descent algorithm that updates each coefficient in turn, holding the other coefficients fixed until convergence is reached. This removes the need to repeatedly manipulate an entire data matrix at once and makes logistic RR feasible even when the data contain hundreds of thousands of predictor variables. The CLG algorithm is described in Supplementary Appendix C.

## Application to Bipolar Disorder Data

We evaluated our method using real SNP data taken from two GWAS of Bipolar Disorder (BD). BD is a complex neurobehavioural phenotype, characterised by episodes of mania and depression. The lifetime prevalence of BD is estimated to be in the region of 1%, and the heritability of BD has been estimated to be as high as 85% [McGuffin et al., 2003]. A number of loci have been identified as associated with BD; however replication studies have not always been successful [Alsabban et al., 2011]. It is thought that many genes of small effect contribute to the liability to develop BD. This hypothesis has been offered as an explanation for the underwhelming findings from BD GWAS [Serretti and Mandelli, 2008].

We used data from the WTCCC [WTCCC, 2007] to fit models to predict BD status. We evaluated these models using an independent test data set, a GWAS of BD from the Genetic Association Information Network (GAIN) [Smith et al., 2009].

Before the model was fitted and evaluated, data were pre-processed and quality control checks were performed following the documented procedures accompanying each data set. Briefly, individuals and genotype calls that had been identified as poor quality by the WTCCC were removed from the data. Missing genotypes were imputed using Impute2 [Howie et al., 2009], with genotypes with the highest posterior probability being used in the analysis. For the GAIN data, only individuals with European ancestry and unambiguous phenotype were used in the test data. Pre-imputation quality control (QC) involved removing one of each of pairs of individuals identified as related in the data, removing invariant SNPs, SNPs with call rate < 98%, and SNPs with Hardy–Weinberg *P*-value 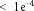. Individuals identified as outliers by the program EIGENSTRAT [Price et al., 2006] were removed from their respective data sets.

Following quality control, the WTCCC data comprised 1,841 cases and 2,886 controls, and the GAIN data comprised 995 cases and 1,025 controls. Following QC, genotypes will still be of variable quality. It would be possible to extend our approach to handle probabilistic genotype scores [Marchini et al., 2007]. In order to evaluate the predictive models, it was necessary to have the same predictors (SNPs) in both the training and test data sets. Approximately 300,000 autosomal SNPs that were common to both data sets were used in the analysis. PLINK v1.07 [http://pngu.mgh.harvard.edu/purcell/plink/, Purcell et al. [2007]] was used for pre-imputation quality control and data preparation steps.

Having obtained directly typed and imputed SNPs such that we had the same SNPs in the two data sets, predictive models were fitted. In these data with a binary outcome, the logistic model was used to describe the relationship between genotypes and disease status.

In performing variable selection followed by multiple regression, instead of including a pre-defined proportion of all predictor variables in the multiple regression model, we chose a significance threshold (*P*-value cutoff) for a variable to be included. We evaluated predictive performance at a range of *P*-value thresholds. The number of SNPs that reached each significance threshold investigated is presented in Table3. Inspection of the SNPs with smallest univariate *P*-values in our results revealed that these are not the top hits reported by the WTCCC [WTCCC, 2007]. Studies have found identified significant associations that are in accordance with our results [Oh et al., 2012]. Oh et al. assumed that the difference between their results and those presented by the WTCCC was due to ‘unreported genotype calling errors’. We make no such assumptions. Our aim in this study was to evaluate the performance of the method we propose when compared to other methods that could be used to fit prediction models using genome-wide SNP data, and we do not feel that this discrepancy between our findings and those of the WTCCC invalidates our results. In HyperLasso regression, as before, we fixed the shape parameter as 3.5 and chose the penalty parameter using 10-fold cross-validation.

**Table 3:** Performance in out-of-sample prediction using Bipolar Disorder data

	Univariate				HL	RR- 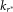
*P-value threshold*	10^−4^	10^−5^	10^−7^	10^−10^		
SNPs reaching threshold	346	58	3	2		
Mean classification error	0.510	0.489	0.491	0.490	0.492	0.465
Brier score	0.35	0.29	0.28	0.26	0.38	0.29

Logistic RR models were fitted on WTCCC-BD data. Mean classification error and Brier score were computed using the GAIN-BD data.

Regression coefficients were estimated using RR with shrinkage parameter 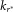. In order to prevent local regions of high linkage disequilibrium (LD) overwhelming the principal components, the training data were thinned to 1 SNP every 100 kb before choosing the number of principal components and computing 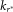. This thinning of the data was evaluated in the simulation studies in the previous section, but thinning did not affect predictive performance in that case (results not shown). Fitted coefficients were subsequently estimated on the full set of SNPs. Results comparing the predictive performance of our proposed method with that of models based on univariate tests of significance and models fitted using HyperLasso regression are presented in Table3. In models fitted using univariate variable selection followed by multiple regression, relaxing the significance threshold for inclusion of a SNP in the model quickly led to more SNPs reaching the threshold than there are observations in the data. With more predictors than observations, a multiple regression model cannot be fitted. Thus when using the univariate variable selection approach, we necessarily discard information in the large number of SNPs that are moderately associated with outcome. HyperLasso regression presents the problem of the choice of the two penalty parameters that control the amount of shrinkage. Choice of the parameters by cross-validation is computationally intensive, becoming unfeasibly so for large data sets such as this one. Our method has the advantage of not requiring cross-validation and offering improved predictive performance. Again we see that our proposed estimator offers good predictive performance in comparison to other regression approaches as well as having computational advantages. Prediction accuracy, measured using the Brier score, is best in a univariate model containing only the most strongly associated SNPs (*P*-value threshold 10^−10^). However, this comes at a cost of a larger mean classification error, and as explained above, discarding many SNPs corresponds to discarding the information in those SNPs.

In Supplementary Fig. S2 we show a receiver operating characteristic curve (ROC curve), plotting true positive rate (TPR) against false positive rate (FPR) as the probability threshold for classification as a case is varied. We see that compared to both HyperLasso regression and multiple regression based on univariate variable selection, our method has higher TPR and lower FPR at all values of the threshold. The ROC curve also demonstrates the challenge of using genetic data to predict disease status.

## Discussion

We have introduced a semi-automatic method to guide the choice of shrinkage parameter in ridge regression. Our method is particularly useful when the regression problem comprises more predictor variables than observations, a situation that often arises in genetic data. This is because existing ridge estimators such as that proposed by Hoerl et al. [1975] cannot be computed in such settings.

Disease risk prediction using genetic information remains a challenging problem due to the high dimensionality and correlation structure of the data. RR is a technique that addresses these difficulties and has been shown to offer good predictive performance. As we demonstrated using a real data example, our method can feasibly be applied to genome-wide SNP data, and this is the setting in which we envisage it will be used. Although whole-genome sequencing of patients is not yet routine in the clinic, SNP genotyping is rapidly decreasing in cost and could feasibly be applied were prediction models available. Among penalised regression methods, RR has been shown to offer good predictive performance. Our method facilitates the application of RR to genetic data for the construction of prediction models.

Our method has computational and practical advantages over competing methods. Because the choice of shrinkage parameter is semi-automatic, our method does not require computationally intensive CV. Nor is determination of causal variables necessary, as is the case when selecting predictor variables to include in a multivariate model fitted using OLSR. Using training data to build a model that is subsequently used to predict on new data relies on the obvious assumption that the two samples are drawn from the same population. The two samples must have in common both the disease phenotype being studied and the genetic architecture in terms of number of causal variables and their effect sizes. This is true of any prediction method and is not a problem limited to our approach. Prediction performance will degrade when this assumption is not met, but it can be a difficult assumption to verify, particularly for disease phenotypes with heterogeneous etiology.

One challenge in the development of prediction models based on case-control data, as in our real data example, is that case-control samples contain an overrepresentation of cases compared to the number in the general population. Hence, population disease prevalence is implicitly overestimated in the fitted model. Methods have been proposed in the literature to adjust the baseline disease risk in the fitted model based on estimates of disease prevalence in the population. Proposed methods include intercept adjusted maximum likelihood estimation [Anderson, 1972; Greenland, 2004] and case control weighted maximum likelihood estimation [Rose and van der Laan, 2008]. Both of these methods are reliant on a good estimate of population disease prevalence, and if such an estimate were available they could easily be incorporated in our framework. We note that performing the same adjustment of baseline risk across all the models being compared would not affect the relative results of the performance comparison.

Using simulation studies, we demonstrate that our method outperforms competing penalised regression methods in terms of prediction error when data comprise more predictors than observations and there are many causal variables with small effects, a situation that is representative of genetic data. We demonstrate the good predictive performance our method by using data from two genome-wide association studies to construct and evaluate a prediction model.

Because RR is a regression approach, the method can be extended to include additional, non-genetic covariates. For example, clinical information or PCs to correct for population structure could be included. It would be possible to extend the approach to investigate higher order interaction terms, albeit at an increased computational cost. Given the large number of predictor variables in a GWAS, it may be necessary to perform a variable selection step before investigating interaction effects.

## Conflict of Interest

The authors have no conflict of interest to declare.

## Supporting Information

Disclaimer: Supplementary materials have been peer-reviewed but not copyedited.
