# Supplementary material for: Ridge Regression in Prediction Problems: Automatic Choice of the Ridge Parameter
Source: Genet Epidemiol. 2013 Jul 26;37(7):704–14. doi: 10.1002/gepi.21750 (PMC4377081; doi:10.1002/gepi.21750)
Supplement: Supplementary file 1 — Supporting Information [file gepi0037-0704-sd1.pdf]

Ridge regression in prediction problems: automatic choice of the ridge parameter

Supporting Information

Erika Cule and Maria De Iorio

Supplementary Tables

Table 1: Four simulation scenarios used in the evaluation of the bias-variance decomposition. The simulation scenarios are taken from Zou & Hastie (2005).

scenario	n	p	$\boldsymbol{\beta}$	Structure of $\mathbf{X}$
(1)	100	8	$(3, 1.5, 0, 0, 2, 0, 0, 0)$	$\text{corr}\,(i, j) = 0.5^{\lvert i-j \rvert}$
(2)	100	8	0.85 for all j	$\text{corr}\,(i, j) = 0.5^{\lvert i-j \rvert}$
(3)	50	40	$\beta_j = \begin{cases} 0 & j = (1, \ldots, 10, 21, \ldots, 30) \\ 1 & j = (11, \ldots, 20, 31, \ldots, 40) \end{cases}$	$\text{corr}\,(i, j) = 0.5$ for all i and j
(4)	50	40	$\beta_j = \begin{cases} 0 & j = (1, \ldots, 15) \\ 1 & j = (16, \ldots, 40) \end{cases}$	$\mathbf{x}_j = Z_1 + \epsilon_j^x, \quad Z_1 \sim \mathcal{N}(0,1) \quad j = 1, \ldots, 5$<br>$\mathbf{x}_j = Z_2 + \epsilon_j^x, \quad Z_2 \sim \mathcal{N}(0,1) \quad j = 6, \ldots, 10$<br>$\mathbf{x}_j = Z_3 + \epsilon_j^x, \quad Z_3 \sim \mathcal{N}(0,1) \quad j = 11, \ldots, 15$<br>$\mathbf{x}_j \sim \mathcal{N}(0,1) \quad j = 16, \ldots, 40$

n, number of observations; p, number of predictors; $\boldsymbol{\beta}$, vector of coefficients; $\mathbf{X}$, matrix of predictors.

Table 2: Performance in out-of-sample prediction in the presence of variance heterogeneity.

	Univariate					HL	RR-k_{r^*}
% of SNPs ranked by univariate p-value	0.1%	0.5%	1 %	3%	4%		
Mean PSE	1.16	1.19	1.21	1.31	1.33	1.12	1.13

HL, HyperLasso regression; RR-k_{r^*}, RR with the shrinkage parameter k_{r^*}.

Supplementary Figures

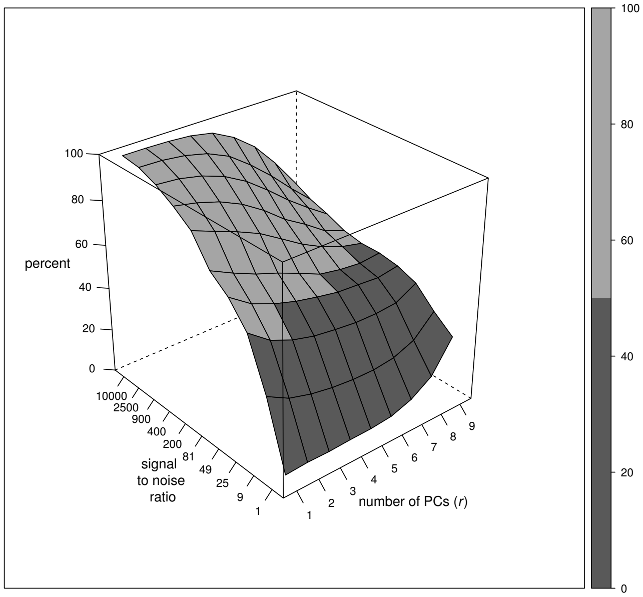

Figure 1: Comparing the mean-squared error of ridge regression estimates obtained using the shrinkage parameter k_r to those obtained using the shrinkage parameter k_{HKB}. Plotted are the proportion of replicates that using k_r results in smaller mean squared error than the estimates fitted using k_{HKB} (equivalent to k_r with $r = p$).

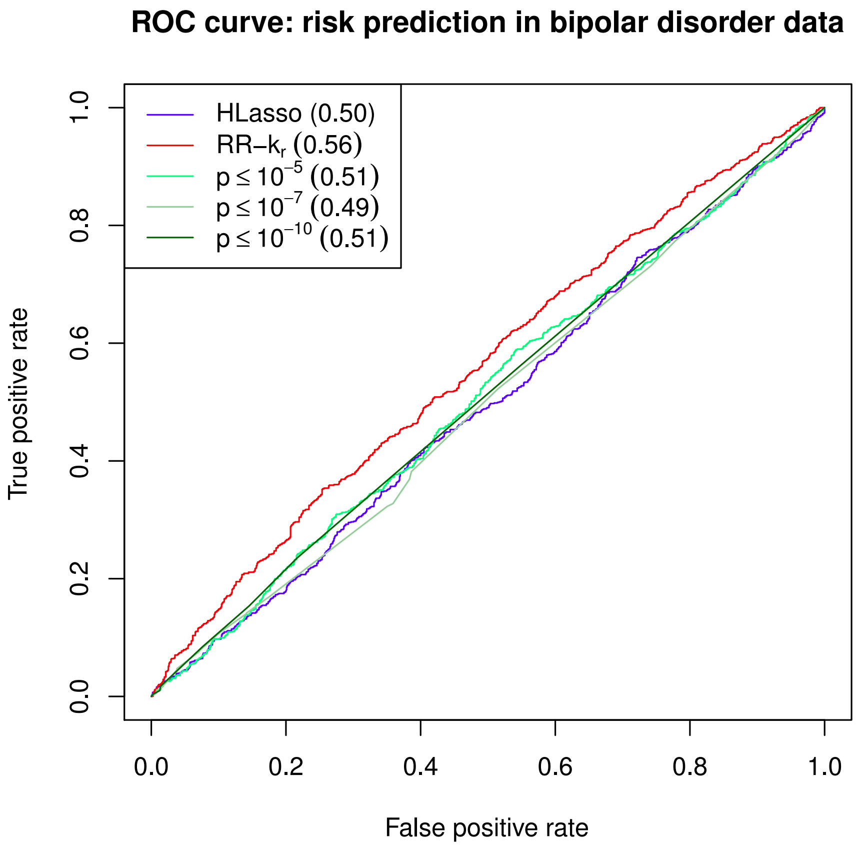

Figure 2: Receiver operating characteristic curve (ROC curve) plotting true positive rate (TPR) against false positive rate (FPR) as the probability threshold for classification as a case is varied. Area under the curve (AUC) statistics are given in the legend, in parentheses. HLasso, HyperLasso regression; RR-k_{r^*}, RR with shrinkage parameter k_{r^*}; univariate significance thresholds are for inclusion of individual SNPs in a multiple regression model. Regression models were fitted on WTCCC-BD data, and evaluated on GAIN-BD data, with the models evaluated on GAIN-BD data plotted here. For details of the study data and methods used to fit the regression models, see main text.

Supplementary Appendix A The number of principal components to include in k_r

We address whether, when computing k_r, it is more useful to include all non-zero PCs (of which there are at most $\min(n, p)$), or to include fewer than all the non-zero PCs. To this end we reanalyse the data analysed by Hoerl et al. (1975), extending their results to compare the shrinkage parameter k_r to k_{HKB}. The data being reanalysed are a ten-factor dataset consisting of 36 observations. These data were first discussed by Gorman & Toman (1966) and are described in Daniel et al. (1999). They relate to the operation of a petroleum refining unit. Following the approach taken by Hoerl et al. (1975), we use the ten-factor dataset as a design matrix in a simulation study. In each replicate, a vector of regression coefficients with a specified squared length is simulated. As in Hoerl et al. (1975) we find that, subject to normalisation, our results are not sensitive to this value. Response variables are simulated at a range of signal-to-noise ratios, where the signal is the squared length of the coefficients used to generate the data and the noise is the error in the responses, σ^2. For each signal-to-noise ratio, 1000 replicates are simulated, and results are reported as an average of these. Hoerl et al. (1975) tabulate the mean squared error under both the linear and ridge models and report the percentage of replicates linear regression gives rise to estimates $\hat{\boldsymbol{\beta}}$ with smaller smaller mean squared error than ridge estimates $\hat{\boldsymbol{\beta}}_{k_{\text{HKB}}}$ with k_{HKB} defined as in Equation (**??**). Following this approach, in Supplementary Figure 1 we plot the percentage of replicates that k_r results in ridge estimates $\hat{\boldsymbol{\beta}}_{k_r}$ with smaller mean squared error than the estimates obtained using the shrinkage parameter k_{HKB}. From this figure we see that, when the signal to noise ratio is not too low, estimates of $\hat{\boldsymbol{\beta}}$ with smaller mean squared error are obtained using k_r with $r < p$ than when using k_{HKB}.

Supplementary Appendix B Definitions of Degrees of Freedom in penalised regression models

Ordinary least squares regression (OLSR), ridge regression (RR) and principal components regression (PCR) all result in models of the form given in Equation (**??**). For models that can be expressed in this form, several definitions of effective degrees of freedom have been proposed (Hastie & Tibshirani, 1990).

The effective number of parameters, $\text{tr}(\mathbf{H})$, gives an indication of the amount of fitting that $\mathbf{H}$ does. As discussed in the main text, $\text{tr}(\mathbf{H}\mathbf{H}')$ can be defined as the effective degrees of freedom for variance. The degrees of freedom for error, is given by $n - \text{tr}(2\mathbf{H} - \mathbf{H}\mathbf{H}')$, thus the effective number of parameters in the degrees of

freedom for error is $\mathrm{tr}(2\mathbf{H} - \mathbf{HH}')$.

In OLSR, RR and PCR it can be shown that $\mathrm{tr}(\mathbf{HH}') \leq \mathrm{tr}(\mathbf{H}) \leq \mathrm{tr}(2\mathbf{H} - \mathbf{HH}')$ (Hastie & Tibshirani, 1990). In OLSR, all three definitions of degrees of freedom reduce to to p, the number of parameters in the model. In PCR, all three definitions reduce to r, the number of components retained in the PCR.

In RR with $k > 0$, the three definitions take values that follow the inequalities

$$\mathrm{tr}(\mathbf{HH}') \leq \mathrm{tr}(\mathbf{H}) \leq \mathrm{tr}(2\mathbf{H} - \mathbf{HH}') \tag{S1}$$

Proof.

$$\begin{aligned}
\mathbf{H} &= \mathbf{X}\left(\mathbf{X}'\mathbf{X} + k\mathbf{I}\right)\mathbf{X}' \\
&= \mathbf{UDV}'\left(\mathbf{VD}^2\mathbf{V}' + k\mathbf{I}\right)\mathbf{VD}'\mathbf{U}' \\
&= \mathbf{UDV}'\left(\mathbf{V}(\mathbf{D}^2 + k)\mathbf{V}'\right)\mathbf{VD}'\mathbf{U}' \\
&= \mathbf{U}\left[\mathbf{D}^2/(\mathbf{D}^2 + k)\right]\mathbf{U}'
\end{aligned}$$

$\mathrm{tr}(\mathbf{H})$ is the sum of the t diagonal elements of $\mathbf{H}$. Each element is less than or equal to 1. $\mathrm{tr}(\mathbf{HH}')$ is also the sum of t diagonal elements, this time of $\mathbf{HH}'$, and each of which is the square of the corresponding diagonal element of $\mathbf{H}$. These diagonal elements each take a value between 0 and 1, thus the sum of their squares is less than or equal to the sum of the original elements. A similar argument holds for the diagonal elements of $2\mathbf{H} - \mathbf{HH}'$, where the sum is greater than or equal to the sum of the diagonal elements of $\mathbf{H}$:

$$\begin{aligned}
\mathrm{trace}(\mathbf{H}) &= \sum_{j=1}^{t} \lambda_j^2/(\lambda_j^2 + k) \quad t = \min(n, p) \\
\mathrm{trace}(\mathbf{HH}') &= \sum_{j=1}^{t} \lambda_j^4/(\lambda_j^2 + k)^2 \\
\mathrm{trace}(2\mathbf{H} - \mathbf{HH}') &= \sum_{j=1}^{t} \lambda_j^2(\lambda_j^2 + 2k)/(\lambda_j^2 + k)^2
\end{aligned}$$

□

Corollary: For a fixed value of the degrees of freedom, $k_{\mathbf{HH}'} < k_{\mathbf{H}} < k_{2\mathbf{H}-\mathbf{HH}'}$ where $k_{\mathbf{HH}'}$ is k such that $\text{tr}(\mathbf{HH}') = r$, $k_{\mathbf{H}}$ is k such that $\text{tr}(\mathbf{H}) = r$ and $k_{2\mathbf{H}-\mathbf{HH}'}$ is k such that $\text{tr}(2\mathbf{H} - \mathbf{HH}') = r$ (for the same value of r in all three cases).

Proof. We seek $k_{\mathbf{H}}$ and $k_{\mathbf{HH}'}$ such that:

$$\sum_{j=1}^{t} \lambda_j^2/(\lambda_j^2 + k_{\mathbf{H}}) = \sum_{j=1}^{t} \lambda_j^4/(\lambda_j^2 + k_{\mathbf{HH}'})^2 = r$$

For each diagonal element $j = 1 \dots t$:

$$\lambda^2(\lambda^2 + k_{\mathbf{HH}'})^2 = \lambda^4(\lambda^2 + k_{\mathbf{H}})$$

Which simplifies to

$$(2 + \frac{1}{\lambda^2})k_{\mathbf{HH}'} = k_{\mathbf{H}}$$

$(2 + \frac{1}{\lambda^2}) > 0$ so $k_{\mathbf{H}} > k_{\mathbf{HH}'}$

An analogous argument shows that $k_{2\mathbf{H}-\mathbf{HH}'} > k_{\mathbf{H}}$. □

The larger the value of k, the further the ridge estimates are from the OLS estimates. This relationship holds when the ridge estimates are returned to the orientation of the data. In RR with $k > 0$, the three definitions of degrees of freedom follow the inequalities given in Equation (S1). For each of the definitions, it is possible to choose the ridge parameter such that the degrees of freedom equal some specified value. Thus, choosing k such that $\text{tr}(\mathbf{HH}') = r$ (among the three definitions of degrees of freedom) results in regression coefficient estimates that are closest to the OLS estimates.

Supplementary Appendix C Logistic ridge regression by cyclic coordinate descent

In this section, we describe logistic RR and cyclic coordinate descent, the algorithm which we use to compute logistic RR coefficients.

In the logistic regression model, let $\mathbf{X}$ be an $n \times p$ matrix of predictors with rows $\mathbf{x}_i = (x_{i1}, \ldots, x_{ip})$, as in the linear regression model (Equation (**??**)). Let $\mathbf{Y} = (Y_1, \ldots, Y_n)'$ be a vector of observed binary outcomes, $Y_i \in \{0, 1\}$. In biomedical data, this setup is common. The outcome variable represents disease status with cases as 1 and controls as 0.

The ith response Y_i is a Bernoulli variable with probability of success $\pi(\mathbf{x}_i)$. The logistic regression model relates the probability $\pi(\mathbf{x}_i)$ that the ith observation is a case to the predictor variables as

$$\Pr(Y_i = 1|\mathbf{x}_i) = \pi(\mathbf{x}_i) = \frac{e^{\mathbf{x}_i\boldsymbol{\beta}}}{1 + e^{\mathbf{x}_i\boldsymbol{\beta}}} \tag{S2}$$

where $\boldsymbol{\beta}$ is a vector of parameters to be estimated. Logistic RR estimates are obtained by maximising the log-likelihood of the parameter vector, subject to a penalty term. The penalised log-likelihood to be maximised is:

$$l(\boldsymbol{\beta}) = \sum_{i=1}^{n} Y_i \log(\pi(\mathbf{x}_i)) + \sum_{i=1}^{n}(1 - Y_i)\log(1 - \pi(\mathbf{x}_i)) - k\|\boldsymbol{\beta}^2\| \tag{S3}$$

The CLG algorithm [Zhang & Oles (2001)] is a cyclic coordinate descent algorithm for penalised logistic regression. The algorithm is described in detail by Genkin et al. (2007). The CLG algorithm is initiated by setting all of the coefficient estimates to an initial value. Then, each coefficient is updated in turn whilst holding the rest fixed. This has the advantage of avoiding the need for the inversion of large matrices. Convergence is checked after each round of updating all of the coefficients.

In the CLG algorithm, cases are code as $Y_i = 1$ and controls as $Y_i = -1$. Finding the updated coefficient, β_j^{new} that maximises the log-likelihood whilst keeping the other parameters fixed is equivalent to finding the z that minimizes

$$g(z) = \left(\sum_{i=1}^{n} f\left(r_i + (z - \beta_j)x_{ij}y_i\right)\right) + \frac{z^2}{2\tau} \tag{S4}$$

where $\tau = \frac{1}{2k}$ and the $r_i = \boldsymbol{\beta}'\mathbf{x}_i y_i$ are computed using the current value of $\boldsymbol{\beta}$ and so are treated as constants.

$f(r) = \ln(1 + \exp(-r))$, and penalty terms not involving z are constant and therefore omitted.

The β_j^{new} that gives the minimum value of $g(\cdot)$ does not have a closed form, so each component-wise update requires an optimization process. Zhang & Oles (2001) use a modification of Newton's method in computing the component-wise updates. The proposed updates are adaptively bounded to prevent large updates in regions where a quadratic is a poor approximation to the objective. Following Genkin et al. (2007) we use as the proposed update:

$$\Delta\nu_j = \frac{\sum_{i=1}^{n}(x_{ij}y_i)/(1+\exp(r_i)) - \beta_j/\tau}{\sum_{i=1}^{n} x_{ij}^2 F(r_i, \Delta_j|x_{ij}|) + 1/\tau} \tag{S5}$$

Genkin et al. (2007) use

$$F(r,\delta) = \begin{cases} 0.25 & \text{if } |r| \leq \delta \\ \frac{1}{2+\exp(|r|-\delta)+\exp(\delta-|r|)} & \text{otherwise} \end{cases} \tag{S6}$$

but other functions can be used (Zhang & Oles, 2001). We then apply the trust region restriction:

$$\Delta_j^{\text{new}} = \max(2|\Delta\beta_j|, \Delta_j/2) \tag{S7}$$

to give the actual update:

$$\Delta\beta_j = \begin{cases} -\Delta_j & \text{if } \Delta\nu_j < -\Delta_j \\ \Delta\nu_j & \text{if } -\Delta_j \leq \Delta\nu_j \leq \Delta_j \\ \Delta_j & \text{if } \Delta_j < \Delta\nu_j \end{cases} \tag{S8}$$

Convergence is declared when $(\sum_{i=1}^{n}|\Delta r_i|)/(1+\sum_{i=1}^{n} r_i) < \epsilon$, where $\sum_{i=1}^{n}|\Delta r_i|$ is the sum of the changes in the linear scores once all the coefficients have been updated, and ϵ is a user-specified tolerance. The CLG algorithm is summarized in Algorithm 1.

References

Daniel C, Wood FS, Gorman JW, 1999. Fitting Equations to Data: Computer Analysis of Multifactor Data. Wiley Classics Library, John Wiley & Sons.

Genkin A, Lewis D, Madigan D, 2007. Large-scale Bayesian logistic regression for text categorization. Technometrics 49:291–304.

Algorithm 1 CLG [Zhang & Oles (2001); Genkin et al. (2007)]

Require: $\beta_j \leftarrow 0, \Delta_j \leftarrow 1$ for $j = 1, \ldots, p$; $r_i \leftarrow 0$ for $i = 1, \ldots, n$
  while do
    for $j = 1 \ldots p$ **do**
      compute tentative step $\Delta\nu_j$ (Equation (S5)).
      $\Delta\beta_j \leftarrow$ (max()) (Limit step to trust region)
      $\Delta r_i \leftarrow \Delta\beta_j x_{ij} y_i$, $r_i \leftarrow r_i + \Delta r_i$ **for** $i = 1, \ldots, n$
      $\beta_j \leftarrow \beta_j + \Delta\beta_j$
      $\Delta_j \leftarrow \max(2|\Delta\beta_j|, \Delta_j/2)$ (update size of trust region)
    end for
    while $(\sum_{i=1}^{n} |\Delta r_i|)/(1 + \sum_{i=1}^{n} r_i) > \epsilon$
  end while

Gorman J, Toman R, 1966. Selection of variables for fitting equations to data. Technometrics 8:27–51.

Hastie T, Tibshirani R, 1990. Generalized Additive Models. Chapman & Hall.

Hoerl AE, Kennard RW, Baldwin KF, 1975. Ridge regression: some simulations. Communications in Statistics - Theory and Methods 4:105–123.

Zhang T, Oles F, 2001. Text categorization based on regularized linear classification methods. Information Retrieval 4:5–31.

Zou H, Hastie T, 2005. Regularization and variable selection via the elastic net. Journal of the Royal Statistical Society B 67:301–320.
